# A Summary of the BRIDGe Summit on Damage-Related Progression of Ulcerative Colitis: Establishing Research Priorities

**DOI:** 10.1053/j.gastro.2022.08.013

**Published:** 2022-08-11

**Authors:** Noa Krugliak Cleveland, Brian Bressler, Corey A. Siegel

**Affiliations:** 1 University of Chicago Medicine Inflammatory Bowel Disease Center, Chicago, Illinois; 2 University of British Columbia, Vancouver, British Columbia, Canada; 3 Gastroenterology and Hepatology, Dartmouth-Hitchcock Medical Center, Lebanon, New Hampshire

**Keywords:** Ulcerative Colitis, Disease Progression, Bowel Damage

## Meeting Summary: Overview

Bowel damage in Crohn’s disease (CD) has been well described, and led to development of the Lemann Index.^1^ However, damage and progression in ulcerative colitis (UC) remain incompletely understood, at least in part because they remain poorly defined. Over the last several decades, a body of evidence has demonstrated that UC leads to alterations in colon structure and function, yet these alterations are not adequately captured by current definitions of disease severity or phenotype, nor are they accounted for in current measures used in clinical trials and clinical practice.^2,3^ Furthermore, it is unclear whether damage in UC occurs rapidly or slowly, whether it occurs in a stepwise fashion, whether it is modifiable or preventable, and whether damage may lead to worse disease outcomes.

In May 2022, the Building Research in Inflammatory Bowel Disease Globally (BRIDGe) Group (www.BRIDGEeIBD.com) convened a 1-day, moderated, hybrid live/virtual roundtable comprising BRIDGe members and additional topic experts designed to address gaps central to advancing the understanding of damage-related progression in UC. This meeting summary presents 8 knowledge gaps and priorities identified by the panel as critical for improving our understanding of the natural history and clinical consequences of progressive UC (Table 1). The group proposes the term *damage* be defined as the accumulation of changes in bowel structure or function, and the term *progression* be defined as a change in the disease state as a result of bowel damage and is associated with worse clinical outcomes.

## Enhance Awareness About Damage-Related Progression of Ulcerative Colitis

Deeper understanding of UC as a disease associated with bowel damage and progression will only be possible with broad awareness of its manifestations and clinical consequences among both clinicians and patients. Among clinicians, this will require an understanding that a healed mucosa may not equate to a healed bowel. It will require us to commit to exploring underlying causes of persistent symptoms beyond our current algorithms and to identify and use new tools to measure these outcomes. Persistent clinical symptoms despite endoscopic remission are often attributed to irritable bowel syndrome without further investigation or adequate understanding of the underlying causes. However, symptoms may be explained by ongoing histologic or molecular disease activity that is not detectable by endoscopy or biochemical markers currently available.^4,5^ Alternatively, alterations to bowel wall layers deeper than the mucosa or dysmotility and/or reduction in bowel wall compliance may be driving these symptoms, and could therefore represent UC-related bowel damage.^6-8^

At the same time, patients will require an understanding that achieving endoscopic remission may not translate into normalization of symptoms and, conversely, that despite improvement in mucosal inflammation or clinical symptoms, bowel damage may have ensued, leading to disease progression and worse outcomes.

## Identify Patients With Persistent Clinical Symptoms Despite Mucosal Healing

A substantial proportion of patients with endoscopic remission continue to show persistent clinical symptoms. Only 30%–40% of patients with UC with mucosal healing report normal stool frequency, despite endoscopic remission.^9^ In fact, this phenomenon of lower rates of clinical remission compared with endoscopic remission is seen across all pivotal clinical trials of biologic therapies in both induction and maintenance of UC.^10,11^ Although we believe this discrepancy points to a need for a more precise measure of disease activity, sizing the proportion of patients that fall under this category may help to clarify the underlying cause. We propose that this should be studied prospectively, while categorizing patients by different definitions of endoscopic healing, that is, a Mayo endoscopic score of 0 or 1 or a Mayo endoscopic score of 0 only, the combined end point of endoscopic and histologic quiescence, and perhaps even histologic normalization. This will help distinguish whether persistence of bowel-related symptoms in patients with a healed mucosa is due to an inadequate definition of endoscopic healing or to other factors not measured by endoscopy or histology contributing to symptoms. Longitudinal follow-up is also of interest, as this may clarify whether there are time lags between endoscopic healing, histologic healing, and symptomatic resolution. Furthermore, in the group of patients in whom symptoms persist despite endoscopic and histologic healing, assessment for transmural bowel damage would be of interest. Selection of proper assessment tools of clinical symptoms, defining appropriate time points for their measurement, and identifying invasive and noninvasive tests that can reliably measure components of bowel damage is critically important. Another key part of this effort will be to identify those who do not experience this discrepancy between clinical remission and mucosal healing, as this will, in turn, be imperative in identifying predictors of progressive UC.

## Define Components of Bowel Damage and Develop a Severity Scale for Each Component

We and others have developed disease severity indices for UC comprising a variety of endoscopic, laboratory, and clinical end points.^12,13^ However, with our current understanding that both clinical symptoms and bowel damage may be present despite endoscopic healing, defining individual components of bowel damage and developing a severity scale for each component is critical to stratifying patients for appropriate management and monitoring over time.

Changes in bowel structure can conceivably be considered components of bowel damage in UC. These may include bowel damage on endoscopy characterized by a tubular/featureless colon, visible scarring, or pseudopolyps. Histologic evidence of disease activity or, conversely, normalization of histology, are other structural changes that can be considered. Radiographically, loss of haustration, as seen in a so-called “lead-pipe colon,” or increased bowel wall or submucosal thickness could also define structural bowel wall damage, which can also be seen histologically in colectomy specimens. Finally, alterations in functional outcomes, such as bowel transit time, motility, or wall compliance should all be included in the definition of bowel damage. Further research is crucial to characterize the spectrum of these changes and longitudinal assessment is needed to characterize the timing and natural history of bowel damage in relation to disease activity, as well as their clinical and functional consequences. Importantly, each of these potential components may be more severe or less severe in any given patient with UC progression, high-lighting the need for an objective severity score for each component. Development of a severity scale will also help define the characteristics of each component and minimize the potential impact of clinician experience on patient assessment.

## Define Measures of Ulcerative Colitis Progression

Strictures, abscesses, and fistulas are well-defined, measurable indicators of damage-related progression of CD.^14^ The challenge in defining measures of UC-related bowel damage and progression is to distinguish between its components and its consequences. Stool frequency, urgency, neoplasia development, impaired quality of life, and disability are likely consequences of progression and may be related to underlying damage. Other features of progressive UC to consider include extra-intestinal manifestations, such as rheumatic, dermatologic, and atherosclerotic diseases, as well as neurodegenerative and mood disorders.

Distinguishing indicators that may not be components of UC progression is equally important. For example, as disease duration does not independently predict rectal compliance, fibrosis, or muscularis mucosa thickening,^7,15^ assessing the effects of progression over time will help in designing strategies for management and prevention.

## Define Phenotypes of Ulcerative Colitis Progression

Behavior of UC is noticeably absent from the Montreal UC classification,^16,17^ suggesting there is a lack of predictability to the way the disease might progress or regress over time. Yet population studies suggest there are multiple phenotypes of disease,^18^ and it is possible that, with improved understanding, patients may be classified with progressive vs nonprogressive UC. There may also be subcategories of these phenotypes, such as early vs late progression, or subphenotypes categorized by different components of progression.

Expert consensus will be required to assign bounding parameters for each phenotype, as well as meaningful clinical outcomes associated with them, so that patients could be categorized and classified according to the different disease behaviors.

## Define Clinical Consequences of Bowel Damage

Inherent in the definition of bowel damage is the assumption that such findings are clinically relevant, that is, that they affect disability and quality of life. Current UC-specific disability scales measure symptoms, such as stool frequency and urgency and rectal bleeding, based on endoscopic disease severity.^19^ Particularly in the presence of endoscopic remission, it will be important to consider the specific effects of bowel damage on these symptoms and, conversely, whether symptoms in patients with progressive UC accurately reflect the underlying disease process. For components of bowel damage that are potentially reversible, such as bowel wall thickness, correlating changes in symptoms as the bowel normalizes will be critical to determining whether this would, in turn, reduce risk for colectomy or neoplasia and improve outcomes overall. Distinguishing symptoms due to bowel damage from irritable bowel syndrome symptoms independent of bowel damage that may be present in patients with quiescent disease^20^ may also be clinically important, and it is possible that investigation will identify drivers of clinical consequences, such as risk for colectomy, dysmotility, poor quality of life, and greater degree of disability.

## Identify and Validate Tools to Measure Bowel Damage

A key challenge in defining bowel damage is our limited ability to measure it in the clinic. To properly assess patients with UC progression, it will be critical to identify and validate tools that can be used outside of academic research settings, that are noninvasive or minimally invasive, and that can help distinguish individual components of bowel damage that most directly affect function and clinical outcomes. Current tools could be modified to target the components of bowel damage seen in UC, and new tools and techniques could be applied on the basis of knowledge already gained from CD and other diseases.

Intestinal ultrasound can visualize the various bowel wall layers in vivo and is becoming a key modality to measure structural bowel wall changes. Other modalities to explore include advanced dynamic imaging studies that can provide measurements of bowel motility and function. Tools to quantify colonic fibrosis, such as shear-wave elastography are of great potential, although shear-wave elastography as used for the quantification of fibrosis in CD has yet to be validated in UC-related colonic fibrosis. Alternatively, circulating serum markers that could quantify colonic fibrosis, such as those studied in liver fibrosis, would be of great interest. The use of a barostat to measure bowel wall compliance or the development of surrogate point of care measures of reduced compliance are additional prospects, as are enhanced endoscopic visualization techniques and the use of artificial intelligence to enhance sensitivity for the detection of microscopic disease activity. This might also include incorporation of single-cell sequencing, considerations for tissue-specific spatial resolution, and other molecular technologies.^21^

Validating such tools would require care to ensure that outcomes from noninvasive techniques correlate with histopathology data, that all measured outcomes can be reliably linked to clinical outcomes and, most important, that changes in bowel damage components that are potentially modifiable can be accurately measured and assessed for reversibility. Once validated, such tools will prove highly valuable for clinicians and the research community.

## Develop and Validate a Ulcerative Colitis Bowel Damage Index

Developing and validating a bowel damage index in UC is the final step to standardize future research and clinical studies of progression in UC. Ideally, such an index could be used to measure cumulative damage at a specific time point, as well as progression of UC over time. This will allow for identification of predictors of bowel damage and stratify patients at high or low risk of progression, compare effects of treatment on UC progression, and highlight opportunities to prevent or reverse further bowel damage. It should account for location and extent of bowel damage to assess its relative clinical importance and incorporate the severity scale developed for each component of bowel damage. It could be limited strictly to anatomic/structural changes directly related to the defined components of bowel damage, or it could be constructed to also consider functional changes and other clinical consequences (such as neoplasia development). The index would ideally be able to distinguish active inflammation that might respond to current therapies from bowel damage that is independent of inflammation, and encompass standardized scoring systems and definitions that can be used in clinical research studies and be readily applied to clinical practice.

Given the broad impact of bowel damage and the limitations posed by available technologies in standard clinical practice, it may prove necessary to develop a set of indices, each focused on a specific end point, such as bowel wall structure, using histologic assessment as the reference standard and endoscopy and intestinal ultrasound as surrogates for this in vivo. In such a case, treatments targeted toward improving this “bowel-wall structure index score” may seek to achieve mucosal healing with the addition of normalizing overall bowel wall and submucosal thickening, and minimizing fibrosis and muscularis mucosal hypertrophy.

## Conclusions

The BRIDGe Summit on damage-related progression of UC was convened to review available evidence and outline the gaps needed to improve our understanding of the natural history and clinical consequences of bowel damage in UC. As a first step, we identified 8 research priorities to guide the field moving forward (Table 1). The next vital steps will be to implement strategies to increase awareness of bowel-damage–associated progression in UC, define components of bowel damage and their clinical consequences, identify the proportion of affected patients, and describe phenotypes of UC that correlate to the degree of bowel damage and disease outcomes. We anticipate that large research efforts will identify the affected population and Delphi expert panels comprising gastroenterologists, neurogastroenterologists, surgeons, radiologists, and pathologists will reach consensus on what to include in each biologic and clinical definition. In the long-term, we will identify and validate tools to measure bowel damage in clinical practice and develop and validate a bowel damage index that can be used to assess and monitor patients’ response to therapy and their disease progression over time.

We recognize that current tools and technologies may limit our ability to fully assess these changes in patients with UC, and that it will likely require many years of effort to accomplish the tasks laid out here. Nevertheless, we note that it took nearly a decade to develop and validate the Lemann Index, which measures cumulative structural bowel damage in CD.^1,14^ Therefore, we identify these research priorities to challenge ourselves and our colleagues to achieve the ultimate goal of predicting, preventing, and reversing progression and bowel damage in UC.

## Figures and Tables

**Table 1.** Research Priorities for Understanding Damage-Related Progression of Ulcerative Colitis

1.	Enhance awareness about damage-related progression of UC
2.	Identify patients with persistent clinical symptoms despite mucosal healing
3.	Define components of bowel damage and develop a severity scale for each component
4.	Define measures of UC progression
5.	Define phenotypes of UC progression
6.	Define clinical consequences of bowel damage
7.	Identify and validate tools to measure bowel damage
8.	Develop and validate a UC bowel damage index

## Abbreviations used in this paper:

BRIDGe: Building Research in Inflammatory Bowel Disease Globally
CD: Crohn’s disease
UC: ulcerative colitis

## References

[1] Pariente B, Cosnes J, Danese S, Development of the Crohn’s disease digestive damage score, the Lemann score. Inflamm Bowel Dis 2011;17:1415–1422. 21560202 10.1002/ibd.21506 PMC3116198

[2] Torres J, Billioud V, Sachar DB, Ulcerative colitis as a progressive disease: the forgotten evidence. Inflamm Bowel Dis 2012;18:1356–1363. 22162423 10.1002/ibd.22839

[3] Krugliak Cleveland N, Torres J, Rubin DT. What does disease progression look like in ulcerative colitis, and how might it be prevented? Gastroenterology 2022;162:1396–1408. 35101421 10.1053/j.gastro.2022.01.023

[4] Christensen B, Hanauer SB, Erlich J, Histologic normalization occurs in ulcerative colitis and is associated with improved clinical outcomes. Clin Gastroenterol Hepatol 2017;15:1557–1564.e1. 28238954 10.1016/j.cgh.2017.02.016 PMC5618439

[5] Rubin DT, Surma BL, Gavzy SJ, Positron emission tomography (PET) used to image subclinical inflammation associated with ulcerative colitis (UC) in remission. Inflamm Bowel Dis 2009;15:750–755. 19090558 10.1002/ibd.20819

[6] Gordon IO, Agrawal N, Willis E, Fibrosis in ulcerative colitis is directly linked to severity and chronicity of mucosal inflammation. Aliment Pharmacol Ther 2018;47:922–939. 29411405 10.1111/apt.14526 PMC5842117

[7] Krugliak Cleveland N, Rai V, El Jurdi K, Ulcerative colitis patients have reduced rectal compliance compared with non-inflammatory bowel disease controls. Gastroenterology 2022;162:331–333.e1. 34597674 10.1053/j.gastro.2021.09.052 PMC8678316

[8] Reddy SN, Bazzocchi G, Chan S, Colonic motility and transit in health and ulcerative colitis. Gastroenterology 1991;101:1289–1297. 1936799 10.1016/0016-5085(91)90079-z

[9] Jharap B, Sandborn WJ, Reinisch W, Randomised clinical study: discrepancies between patient-reported outcomes and endoscopic appearance in moderate to severe ulcerative colitis. Aliment Pharmacol Ther 2015;42:1082–1092. 26381802 10.1111/apt.13387 PMC5049645

[10] Feagan BG, Rutgeerts P, Sands BE, Vedolizumab as induction and maintenance therapy for ulcerative colitis. N Engl J Med 2013;369:699–710. 23964932 10.1056/NEJMoa1215734

[11] Rutgeerts P, Sandborn WJ, Feagan BG, Infliximab for induction and maintenance therapy for ulcerative colitis. N Engl J Med 2005;353:2462–2476. 16339095 10.1056/NEJMoa050516

[12] Siegel CA, Whitman CB, Spiegel BMR, Development of an index to define overall disease severity in IBD. Gut 2018;67:244–254. 27780886 10.1136/gutjnl-2016-312648

[13] Chen G, Lissoos T, Dieyi C, Development and validation of an inflammatory bowel disease severity index using us administrative claims data: a retrospective cohort study. Inflamm Bowel Dis 2021;27:1177–1183. 33043982 10.1093/ibd/izaa263 PMC8314106

[14] Pariente B, Torres J, Burisch J, Validation and update of the lemann index to measure cumulative structural bowel damage in Crohn’s disease. Gastroenterology 2021;161:853–864.e13. 34052277 10.1053/j.gastro.2021.05.049 PMC8609534

[15] Gordon IO, Abushamma S, Kurowski JA, Pediatric ulcerative colitis is a fibrotic disease and linked with chronicity of inflammation. J Crohns Colitis 2021;16:804–821. 10.1093/ecco-jcc/jjab216 PMC9228908 34849664

[16] Silverberg MS, Satsangi J, Ahmad T, Toward an integrated clinical, molecular and serological classification of inflammatory bowel disease: report of a Working Party of the 2005 Montreal World Congress of Gastroenterology. Can J Gastroenterol 2005;19(Suppl A): 5A–36A. 10.1155/2005/269076 16151544

[17] Satsangi J, Silverberg MS, Vermeire S, The Montreal classification of inflammatory bowel disease: controversies, consensus, and implications. Gut 2006;55:749–753. 16698746 10.1136/gut.2005.082909 PMC1856208

[18] Solberg IC, Lygren I, Jahnsen J, Clinical course during the first 10 years of ulcerative colitis: results from a population-based inception cohort (IBSEN Study). Scand J Gastroenterol 2009;44:431–440. 19101844 10.1080/00365520802600961

[19] Bewtra M, Brensinger CM, Tomov VT, An optimized patient-reported ulcerative colitis disease activity measure derived from the Mayo score and the simple clinical colitis activity index. Inflamm Bowel Dis 2014;20:1070–1078. 24810138 10.1097/MIB.0000000000000053 PMC4137887

[20] Gracie DJ, Williams CJ, Sood R, Negative effects on psychological health and quality of life of genuine irritable bowel syndrome-type symptoms in patients with inflammatory bowel disease. Clin Gastroenterol Hepatol 2017;15:376–384.e5. 27189912 10.1016/j.cgh.2016.05.012

[21] Denson LA, Curran M, McGovern DPB, Challenges in IBD research: precision medicine. Inflamm Bowel Dis 2019;25:S31–S39. 31095701 10.1093/ibd/izz078

